# Qualitative analysis to explore the barriers and facilitators around the implementation of automated dispensing cabinets at a large NHS Trust in England

**DOI:** 10.1016/j.rcsop.2025.100562

**Published:** 2025-01-07

**Authors:** Melanie Dalby, Ali Alazab, Navila Talib Chaudhry

**Affiliations:** a King's College Hospital NHS Foundation Trust, Denmark Hill, London SE5 9RS, United Kingdom; b King's College Hospital, School of Cancer & Pharmaceutical Sciences, Franklin-Wilkins Building, 150 Stamford Street, London SE1 9NH, United Kingdom; c Research Department of Practice and Policy, UCL School of Pharmacy, 29-39 Brunswick Square, London WC1N 1AX, United Kingdom

**Keywords:** Automated dispensing cabinets, Technology, Medication supply

## Abstract

**Background:**

Automated dispensing cabinets (ADCs) as a tool to store and manage medication are becoming more widely used in healthcare settings. Although there is literature surrounding their effectiveness at reducing medication error and time for nursing and pharmacy staff, there is little information on how to implement ADCs on a large scale in a busy working clinical environment. The purpose of this study was to qualitatively determine the enablers and barriers to the implementation of ADCs in a hospital setting.

**Methods:**

Participants were selected through purposeful sampling and invited to attend online focus groups and interviews via Microsoft Teams. These were recorded and transcribed. Two facilitators independently reviewed the transcriptions, coded and developed themes using Nvivo.

**Results:**

There were 18 participants that took part in four focus groups and three interviews. These participants were from the pharmacy department, nursing, estates and the external porter company used for medication and supply. Nine themes and 31 sub-themes were identified from the data. The nine themes were; overall thoughts, strategy of implementation, stakeholder engagement, training, workflow changes, environment, evaluation, challenges and solutions to challenges.

**Conclusion:**

This study has provided new insight into the required elements for implementing a large scale piece of new technology into a hospital setting. The data complemented other studies such as the challenges with staff training and the importance of ongoing optimisation of the ADCs post implementation. Key recommendations for others looking to implement ADCs include utilising videos and written materials for training early on and including nursing staff in the core project implementation team as well introducing mock cabinets for staff to practice on.

## Highlights

• Implementation of automated dispensing cabinets in healthcare needs careful planning and consideration of stakeholders.
• Identified challenges were training issues and the management of controlled drugs within automated dispensing cabinets.
• Participants highlighted solutions to enable others to successfully implement technologies in their organisations.

## 1 Introduction

Automated dispensing cabinets (ADCs) containing medication not only improve the security of medicines but also have been shown to reduce medication errors and time for nursing and pharmacy staff.^1^, ^2^, ^3^, ^4^ ADCs are storage devices containing software that is programmed to automate orders and track the distribution of medication.^5^ ADCs use a form of automatic identification and data capture (AIDC) to process medication orders and link to electronic prescribing systems.^5^

The use of ADCs is still a growing trend globally despite being first used in the late 1980s.^6^ Results of the prescribing and transcribing survey conducted by the American Journal of Health-System Pharmacy reported that there are 490 patient care units across the United States that are using ADCs to manage controlled drugs.^7^ In the UK, although the use of ADCs is still not widespread, this is expected to grow especially as nearly half of the National Health Service (NHS) now have electronic prescribing and medicines administration (EPMA) systems.^8^

Even with the growing use of ADCs the volume of literature is still relatively low particularly on the process of implementing ADCs on a large scale in healthcare settings especially in the UK. The institute for safe medication practices produced a guideline for the safe use of ADCs in 2019,^6^ which provides details on nine core safety processes that should be carefully considered prior to implementing ADCs. The guideline highlights some of the reasons why challenges can present and explains that one of the major risk associated with ADCs is the inappropriate use of medication overrides resulting in incorrect selection of drug.

A specific benefit that ADCs bring is a reduction in administration time. Douglas et al. (2017)^9^ report a 40 % reduction in administration time for nurses. Another noted benefit in the literature is a decrease in the number of urgent drug requests to pharmacy and the number of returned products after implementation of ADCs^10^; potentially releasing time for both nursing and pharmacy staff to concentrate on direct patient care and improve efficiency on inpatient wards.^11^ A study conducted at a 450-bed university hospital in Australia captured the experience of nursing and pharmacy staff during the implementation of ADCs.^12^ They identified that the training provided for nurses was insufficient and that pharmacy staff reported a better satisfaction with the implementation. They also reported that work productivity for nursing was impacted due to nursing staff creating workarounds to maximise their efficiency when using the ADCs. A study completed in the same hospital in Australia showed that ADCs negatively impacted nursing workflows by delays in access to the ADC, and increased distances walked by nursing staff to access the ADC.^13^ Balka at al (2007)^14^ found that the volume of discrepancy reports produced by the ADCs was difficult to manage by the resources within the pharmacy team and this could lead to compromised safe medication practices.

The aim of this study is to establish any barriers and facilitators to the implementation of ADCs in a hospital group made up of two sites in the UK.

## 2 Method

### 2.1 Setting

This study was conducted in two hospital sites within the same NHS hospital Trust with a combination of over 900 beds treating one million patients every year.^15^ The larger of the hospital sites is based in London with approximately 54 inpatient wards. The second smaller site is a district general hospital with approximately 30 inpatient wards situated just outside of London. ADCs were implemented at both sites between February 2023 – January 2024 using a two-phase approach. The first phase covered implementation at the larger site followed by implementation at the smaller site.

### 2.2 Data collection

This study was an observational cross-sectional design using focus groups and qualitative interviews as the primary data collection method. Qualitative semi structured interviews and focus groups were used to allow the application of grounded theory, where the data determines the key themes. Qualitative methods were essential to understand the nuanced challenges and successes of ADC implementation and allowed the facilitators to ask probing questions. The use of a survey would not allow the key themes to arise based on the participants response due to the use of structured questions. Interviews were only conducted with participants who were unable to attend a focus group; ensuring we were able to capture all perspectives hence reducing bias in our data collection. A topic guide was produced and reviewed by all authors before being submitted as part of the ethics review. The topic guide contained fourteen open ended questions and was used for the focus groups and the interviews. The literature was utilised to identify suitable questions specifically the ISMP guideline on the safe use of ADCs.^6^

A total of four focus groups and three interviews were conducted (the first two authors were present in the first three focus groups and one interview, and the final focus group and two interviews were conducted by the first author alone due to availability). The first facilitator led the sessions and the second facilitator ensured the session was kept to time. Each focus group and interview took place on Microsoft Teams. This allowed for attendance at the meeting from participants based at different hospital sites. The recording and transcription functionality were utilised.

Full ethical approval via the Health Authority Approval (HRA) was not required as the study was deemed a service evaluation as per the HRA decision tool.^16^ Local ethics approval was sought and gained via the University College London (UCL) research and ethics committee and via the research and audit group at King's College Hospital NHS Foundation Trust (KCH) pharmacy department.

### 2.3 Participants

A purposeful sampling method^17^ was chosen to capture all participants working at KCH who were part of the implementation process of ADCs. A list of participants was created from the core project team. This was used to invite participants and included all involved in the implementation process. A snowballing effect was used whereby participants recommended other participants to attend future focus groups. These extra participants were only included if they fit the criteria of taking part in the implementation process of the ADCs. The snowballing effect only identified a further 3 participants, 1 of which did not respond to the invite, the remainder of which had already been identified. Participants were identified from a variety of departments around the hospital including nursing, pharmacy, information technology (IT), estates and the external medication porter and supply team. Participants were invited via email and were provided with a participant information sheet. The proposed number per focus group was 3–6 per group.^18^, ^19^, ^20^ Participants were consented on the basis of attendance at a focus group or interview.

### 2.4 Data analysis

The transcriptions were downloaded from Microsoft Teams and verified by the first two authors. Where there was uncertainty between the transcription and the recording in a couple of cases, the participant was asked to verify what was said.^21^ The transcriptions were stored on a password protected computer. NVivo version 14.23.3(61) was used to analyse the data by thematic analysis. The second author coded the data according to themes and sub-themes. The first author did the same independently. The final themes, sub-themes and article revisions were reviewed by all authors. Where there were differences, the first and second author reviewed the codes and themes again together to come to an agreement. The third author provided a review and decision for any discrepancies. There were minimal discrepancies but discussions occurred concerning the final naming of some of the main themes.

## 3 Results

In total forty people were invited to attend a focus group. Nineteen people (48 %) accepted but one was unable to attend on the day therefore eighteen (45 %) participants contributed to the data collection. There were four focus groups and three interviews. The participant's gender and discipline are provided in Table 1. Those that were part of the core project team for implementation are stated as such.

**Table 1:** Participant gender and discipline.

Gender	Discipline	Participant ID
Female	Pharmacy	Participant 1 (project team) Participant 2 (project team) Participant 3 (project team) Participant 4 Participant 5 (project team) Participant 6 Participant 7 Participant 8
Male	Pharmacy	Participant 9 (project team) Participant 10 (project team) Participant 11
Female	Nursing	Participant 12 (project team) Participant 13 (project team) Participant 14
Male	Estates	Participant 15
Female	External medication porter and supply team	Participant 16 Participant 17
Male	External medication porter and supply team	Participant 18

Focus group durations ranged from 67 min to 118 min, while interviews lasted 27 min to 47 min.

The data were coded into nine themes and 31 sub-themes. Key recommendations for implementing ADCs identified from the participants can be found in the supplementary section (Table 1 supp).

### 3.1 Theme 1: Overall thoughts

The majority of the participants were positive about the implementation of the ADCs and thought it was well organised. There was a consensus that the lead members of the project team did a good job at driving the project forward. One participant described the project as ‘an unstoppable train’ and that ‘there was, I think, definitely room for improvement if they were going to do this again’ (participant 16). They were referring to the fact that there was little time to stop and reflect which would have allowed for improvement and adjustments to implementation to be made during the process.

#### 3.1.1 Initial days after ADC placement

Many of the participants described the initial days of implementation as challenging. This was due to difficulties in design planning and contacting the correct people to solve earlier issues. One of the lead nurses explained that some of the nursing staff found the change in workflow and practice a struggle in the initial days of usage:

‘The implementation of the [ADC] cabinet within the wards from nursing was a bit of a struggle to begin with … because it was a complete change in practice’ (participant 14).

#### 3.1.2 The success of the implementation

The opinion of whether the implementation has been a success or is still a work in progress split the participants. Participant 9 explained that in general the implementation was successful but at a later point in the focus group the participant then went on to say, ‘it's probably too early to look at’. This suggests that a wider evaluation piece needs to be done at a later point in time to capture the positive or negative changes that ADCs have impacted.

Participant 6 thought that ‘the planning before the implementation and the rollout is the bit I think that could have been better.’ Participant 8 stated that she thought ‘the roll out at [one site] was much more successful.’ This is likely due to the learning that occurred during the roll out at the first site which provided a more effective roll out at the second site.

There were several activities that occurred on the day that an ADC is placed in its designated area as described by participant 5 below:

‘The cabinet is taken up the day before … so the nurses can practice on … make sure that everybody have access and all that, and then go live usually will take the whole day because you have to move the drug from the cupboard into the [ADC]’ (participant 5).

### 3.2 Theme 2: Strategy of implementation

The strategy to implement the ADCs across the two major sites of the organisation involved a number of steps and stakeholders. There was a key element of staff engagement in the area where the ADCs were to be placed, preparing of the room to be ready for placement, the delivery and transfer of medication and then the training and support of staff after placement. The areas that were chosen to implement first where those areas that had ADCs already in place for holding consumable items.

#### 3.2.1 Using a staggered approach

The approach was described as ‘staggered’ whereby ADCs were rolled out to areas ‘step by step’ (participant 5) over a period of time. One hospital site was completed first before moving onto the second site.

#### 3.2.2 Communicating the implementation plan to stakeholders

Communication to the stakeholders ahead of the implementation of any of the ADCs seemed to be well advertised. A member of the project team explained that ‘we made sure the wards were well aware when their [ADCs] were coming. I also checked the week before to make sure they were available for it and the day before as well’ (participant 10). Participant 10 was very keen to ensure the nursing staff were prepared for the arrival of the ADC.

### 3.3 Theme 3: Stakeholder engagement

All participants recognised the importance of engaging the right stakeholders at the right time. A few participants who were not part of the project team stated they ‘were left off of meetings or not engaged early enough in all of the decision making’ (participant 16).

#### 3.3.1 The project team

Participants agreed the project team ‘did well for the pressure that they were under’ (participant 16). Participants were realising of the fact that the project team had deadlines to adhere to and therefore were cognisant of the pressures. Members of the project team themselves admitted that they were satisfied with their achievement by saying; ‘as a team we did really well’ (participant 13).

#### 3.3.2 Who to engage with

The participants were in agreement regarding who the right stakeholders were. These were nursing, including matrons and heads of nursing, pharmacy, IT, estates, the external medication porter team, the ADC manufacturing company along with the pharmacy governance medicine safety team and the hospital quality assurance team. One participant explained that they completed a communication plan to identify ‘my stakeholders, who is going to be affected at what point and within the project and overall that kind of helped’ (participant 13).

#### 3.3.3 Timing of engagement

There was recognition that with a large project that has so many stakeholders, it is difficult to know when each stakeholder group needs to be included. However, participant 15 did state ‘if there was more of an effort to get us involved from beforehand, we could have planned it before … we could have maybe made things more efficient.’ This participant felt that they were not included at the right time.

#### 3.3.4 People on the floor are the key stakeholders

The participants agreed that ‘the key stakeholders really are the people who use it [ADCs] every day and I think we needed more of them in the conversations’ (participant 8). Participant 8 is referring to nurses and is suggesting that more nurses were required as stakeholders.

### 3.4 Theme 4: Training

The main groups of staff that were identified for training were nurses, pharmacy staff and the external medication supply and porter team. One participant highlighted that in some cases the training was delivered too early and that refresher training was needed.

#### 3.4.1 The cascading approach

A cascading approach to training was used due to the number of people that required training. A selection of people called super users received intense training on the use of ADCs and it was the responsibility of these to train others who would then go on to train more staff members.

‘The model that we relied on was super users and a cascading model’ (participant 8).

‘Then the onus was on the super users to disseminate that training down to the end users’ (participant 2).

‘The kind of cascade down, that's almost seen as being the best option because it's so difficult to find other ways of releasing staff’ (participant 11).

#### 3.4.2 Engagement in training

Participant 5 explained that the pharmacy team were not as engaged in training attendance compared to the nursing team. Having a matron or education nurse engaged made it easier to engage with nursing teams as described by the quote from participant 5:

‘Once we have them [matron or education nurse] on board, they will allocate time for the staff member to attend those training, but pharmacy was a bit more challenging’ (participant 5).

#### 3.4.3 Digital literacy

Many of the participants identified that younger staff members found the new software easier to use than older staff members.

‘For young people using, you know, computers [it] is very straightforward. It's easy to, to get your head around, but for older staff member, for older nurses, it was a bit more of a challenge’ (participant 5).

#### 3.4.4 MedX training

MedX is a software system that manages controlled drug stock. Many participants stated that the training for this system was insufficient and was difficult to grasp.

‘It's a hard concept to grasp, so I think adding that into the mix, it makes it hard to train people, especially in areas like A&E (accident and emergency), trauma ward where you've got fast turnover of staff’ (participant 6).

### 3.5 Theme 5: Workflow changes

The introduction of ADCs changed a significant number of workflows for a variety of staff groups. This involved changes during and after the process of implementation.

‘We might have had to get people in overtime on a Saturday to do some of the work. Simply because we couldn't fit into the, the BAU [business as usual] work’ (participant 17).

‘There was the introduction of a new team … new portering team who took, who almost replaced the stores team and took responsibility for putting medicines away’ (participant 9). The introduction of a new portering team to deliver and put away stock medication was a new process for the nursing and pharmacy teams.

#### 3.5.1 Changes to controlled drug management

There were changes to both nursing and pharmacy workflows with regards to controlled drugs. Pharmacy participants explained that the process of dispensing controlled drugs became more lengthy because of the need to print orders and request signatures as per legal regulations. From a nursing point of view, where it is the responsibility of a nurse to count each controlled drug on the ward every night, this process changed after the introduction of the ADCs.

‘We used to … count whatever is left after you've taken the medication. This was the main difference, you [now] have to count before you actually touch or remove any of the drug’ (participant 14).

#### 3.5.2 Changes to medication delivery and administration

The ADCs have allowed for automation of drug ordering which has stopped the requirement of pharmacy staff physically coming to the ward and completing what is known as a ‘drug top-up’. This is where a staff member counts the number of drugs required to re-fill the drug cupboard. With regards to medication administration, initially there were delays to administering medicines as the nurses were queuing to access the ADC to collect medication requested. However, the use of Anywhere RN, a software that allows nurses to order their patients medications from the ADC remotely, reduced the need to manually input the request for medicine to be released from the ADC.

‘You could line up all your drugs, and then you just get to the cabinet and you just boom, boom, boom … they could queue the drugs from the computer they were sitting on in the corridor’ (participant 14).

#### 3.5.3 Effect on staff

One participant described the effect on staff ‘like a change in culture … because nurses had to adapt to new practice’ (participant 13). Other changes such as the introduction of the external medication supply porter team had less impact, ‘I think the transition overall was smooth’. (Participant 17).

### 3.6 Theme 6: Environment

This theme relates to the environment where the ADCs were placed such as in clinical rooms on the ward and the internal configuration of the ADCs themselves.

#### 3.6.1 Issues with room space

Room space was often an issue. In some cases the rooms had to be refitted or an alternative room was used and on occasions door hinges had to be removed to allow the ADC to be moved into the room.

‘The size of the space that you're putting these into in and the location of them really does need to be considered’ (participant 16).

#### 3.6.2 Issues with moving and installation of the ADCs

Issues were raised with regards to moving the ADCs into place as they are heavy and bulky. It was recognised that it is a working hospital, ‘the challenges that we had in clinical ward locations was that you know it's a hospital, it's 24/7 and 365 days a year, nothing closes’ (participant 2). Spacing for storing the ADCs prior to implementation was also raised as the first hospital site had a large building to house the ADCs in, the second site however did not have this space therefore ADCs had to be delivered in batches.

#### 3.6.3 Configuration of the ADC

Careful consideration needs to occur with the placement of the medication within the ADC.

Participant 3 explained, ‘I have also seen a few nurses sometimes struggle to reach the top shelf and we intentionally configure the cabinets to avoid having heavy things high up’.

Lastly, liquid medications in the ADCs have been problematic due to leakage, ‘the liquids have to lie horizontally and that's a big issue, especially around liquid CDs [controlled drugs]’ (participant 4).

### 3.7 Theme 7: Evaluation

The evaluation of the ADCs meant different things to different participants. For example, participant 15 thought about it from a testing of the hardware point of view, ‘For me, it was the testing the, the power sockets … have they been wired back to the board correctly?’ Participant 18 regularly reviews the ADCs for issues as they [estates staff] were being called out to fix issues, ‘I was being called out [for] all sorts of issues. This is why I bought in this, this planned maintenance that I do.’

The project team were praised because ‘they took on a significant proportion of those incidents … like went to the wards to go and find out what happened … they would check like all, all the systems’ (participant 6). Participant 6 felt that some of the incidents that occurred could have been managed by other teams but they were impressed that the project team took on a lot of the incidents.

### 3.8 Theme 8: Challenges

All of the participants raised challenges with regards to the implementation of ADCs. These were often repeated by multiple participants such as the challenges with training. A couple of participants explained that there was inadequate involvement in the consultation process prior to implementation as discussed within the stakeholder theme. The other major challenge of note was the fact that a new electronic prescribing system was implemented during the period of time when the ADCs were being rolled out.

#### 3.8.1 Budgeting

Participant 17 explained that ‘I don't feel the overall cost ends were considered’ and although ‘there's not an endless pot of money to dip into, but I don't feel like the, the correct funding or potential for funding was allocated.’

#### 3.8.2 Issues with communication and conflict

Communication to those staff who needed to know the change was happening was a struggle. Some of the participants raised issues with conflict during the implementation process, ‘I do think some of the issues came when conflict of interest and maybe sometimes because there was so many things going on, the stress can kind of sometimes get to us’ (participant 15).

#### 3.8.3 Issues with controlled drugs

The participants raised a number of challenges with regards to using the ADCs to store controlled drugs. One participant in particular was quite clear that the controlled drugs should not have been put into the ADCs from the beginning:

‘I think [the] launch should have just been for medications with no change to CDs (controlled drugs). I think it was too much of a change to go from cupboards to [ADCs] plus CDs and [ADCs]’ (participant 6).

#### 3.8.4 Issues with resources

Staffing levels, time, funding and also space to house the ADCs prior to implementation were all raised as factors that negatively impacted the implementation. For example, ‘you know in terms of resources in [the] NHS we, we don't always have a lot of time. We don't have a lot of finances to finance what we require’ (participant 13).

#### 3.8.5 Issues with training and support

The challenge with training that participants identified was the volume of staff that needed to be trained and the timing of the training. This put pressure on the super users and the project team to support the end users.

‘It's that retention of training and timing of it's probably really difficult because you have to get everyone ready and but yeah, how to keep it fresh is a, is a challenge’ (participant 1).

#### 3.8.6 Project planning and contingency

It was highlighted that a more formal documented project plan should have been put in place including elements such as a risk register. It was unclear whether or not a risk register was in place or whether it was not well communicated.

‘When we started the project, nobody really looked at the risks. There was nothing really that was put in place to say, OK, if this risk was to happen, how would we mitigate it?’ (participant 13).

#### 3.8.7 Malfunctions

The final challenge sub-theme relates to malfunctions of the ADCs themselves. Participants reported issues with the report printer component of the ADCs, some of the door hinges slipped over time, and where staff were not aware that the fridge needed to be opened via the ADC, there were incidents where the fridge door was forced open thus damaging it.

### 3.9 Theme 9: Solutions to challenges

Although a number of challenges occurred during the implementation the participants did have examples of where these had been resolved or mitigated.

#### 3.9.1 Changes to training

Initially the training was delivered in person to train super users. Over time, after the implementation of the ADCs, training modules were developed including videos and standard operating procedures made available on the hospital intranet pages. A guide was also developed for nurses on the ward to refer to. These were provided for new members of staff as the project team were to be disbanded after the implementation and would not be able to provide face to face training. Ward teams were supported in person on the day of go live from as early as 7:00 am to 9:00 pm.

‘We also did things like we'd go on ward rounds or at least have a wander around the, all the main, major wards at least once a day while we're going through it so we were very visible’ (participant 10).

#### 3.9.2 Communication and meetings

Although it has been recognised that correct communication is very difficult in a large scale project, the participants knew that it was key and there were lessons learnt that led to improvements. For example, ‘the regular team meets are also very useful because it was almost a forum for us to always share what the problems are being encountered and collectively we would look to seek what the best way of actually resolving it would be’ (participant 1). During the initial implementation these were undertaken weekly by the core project team. Participant 1 also stated that ‘the team did a really good job in the internet pages and putting information on there using live links.’ This showed that the project team were good at utilising different types of methods for communicating to get their information across to as wide an audience as possible.

#### 3.9.3 Documentation and written policy

The project team were good at documenting progress and issues throughout the course of the implementation. A central document was kept to record all the issues that occurred. This would then help with duplicate issues as they were able to refer back to the document for solutions. Policies were written ahead of time where possible which supported process issues at later dates. The project team were good at documenting particularly the preparatory meetings which helped the team weeks later to identify who was aware of what and where responsibilities lied. ‘All the conversation that was had in those preparatory meetings were all written and documented’ (participant 2).

#### 3.9.4 Representation from different departments

A member of the project team recognised that their role was to bring people together.

‘When we started we were not really interconnected as the project team, our job was to kind of bring everyone together’ (participant 13). This supports the importance of bringing the right stakeholders together.

Several participants stated that having nursing and senior nursing input helped to drive engagement but also support escalating issues through the appropriate channels.

#### 3.9.5 Supporting staff

The project team worked shifts in line with the ward nursing staff to ensure nurses were supported. The project team and senior nursing staff were available on the ward during the day of go lives to support staff. This meant they were able to support with any training issues or ADC discrepancies.

‘Just spending the extra time rather than anything else, I don't think we did anything else apart from the extra, you know, little teaching sessions’ (participant 14).

#### 3.9.6 Technical solutions

The project team had access to a mechanical officer Monday to Friday for any hardware related issues. Mock cabinets were also utilised to help staff get used to ADCs. These were used in the period between the estates team finishing their work and the actual ADC being placed. Introduction of the use of barcode scanning when putting drugs away in the ADCs supports the external medication supply and porter team.

‘When the restock is done, they will scan the item and then the light will show where the item needs to be put’ (participant 5). If the barcode did not work, the medication supply and porter team implemented a reporting mechanism which automatically sent an email to the project team to resolve.

## 4 Discussion

The aim of this study was to determine the enablers and barriers that staff, who were part of the implementation process of ADCs, identified throughout the project.

The results of this study suggest that the overall implementation of the ADCs across both sites of the hospital group was successful as determined by the participants. This was largely determined by the fact that the ADCs are in place and are functioning to provide medicines at request to nurses on the wards. There are a number of learning points that can be taken forward for new large-scale projects or for other healthcare organisations looking to implement ADCs. However, the long-term success of the ADC would need to be assessed. The initial days of when the ADCs were first in use were difficult, but this was transient as staff became more familiar with their use.^22^

The project team responsible for implementation had a chosen strategy of a staggered approach which allowed for all the elements to come together to enable the placement of the ADC. These elements included the communication to the staff in the area where the placement of the ADC was to happen, the estates team reviewing the area and organising any environment changes, a review process and then placement. Following the placement of the ADC, the medication then had to be transferred into the ADC. The communication by the project team was conducted well during this time. It was noted that the strategy was more successful at the second hospital site as learning was taken from implementation at the first hospital site.

Participants were aware which stakeholders needed to be involved in hindsight. The challenges arose when some stakeholders were not engaged at the correct time or engagement did not carry through the entirety of the project. The lack of engagement in the training from the pharmacy staff, identified by the participants, may have been due to the project team spending more time on engaging staff outside the pharmacy department. For future implementations, it is important to give each stakeholder group equal effort for engagement activities.

The model for training via super users was efficient in that it meant the project team could focus on training a smaller group of staff members. This worked well, however participants expressing selection of correct timing for training, refresher training sessions is supported by Craswell et al. (2021).^12^ The nurses had to change their practice with regards to controlled drug counting. Where before they would count what was left after taking what was required, they are now asked to count what is there before taking anything away. The MedX system within the ADC was difficult to grasp and many reported that the training for this was not sufficient.

As with the implementation of any new technology there are likely to be significant workflow changes. The main changes identified were with the management of controlled drugs,^12^^,^^23^ the medication delivery and ADC filling through a new external porter and supply team and then the changes nurses had with drug administration.^24^

It is clear from the environment theme that careful consideration needs to occur with regards to the placement of an ADC. Some of the rooms needed significant rearrangement disrupting the clinical activity occurring in that area at the time. Configuration of the ADC in terms of where specific medication such as heavy items and liquid bottles need to be planned in advance.^22^ Providing ideal environmental conditions is one of the core safety process identified in the guidelines for the safe use of automated dispensing cabinets written by the ISMP (2019).^6^

A number of studies have identified the requirement to regularly evaluate and maintain technology implementations.^12^^,^^25^^,^^26^ For example, Cresswell et al. (2020)^27^ have developed a framework to guide implementation and optimisation of the functionality of a health information technology system. Although the project team have not implemented an evaluation approach as formal as this, there was an element of evaluation in terms of the estates team reviewing and critiquing their own work. Another example is the medication and supply team undertaking a regular maintenance of the ADCs to check for malfunctions thus providing a solution to mitigate these challenges. Oludapo et al. (2023)^28^ discuss the problem of how organisations are expected to measure the success of their digital transformation. Although they recognise this challenge they state that evaluation should be a vital feature of the implementation and attention to the normalisation process beyond implementation is critical.^28^

The participants were forthcoming with challenges that occurred throughout the implementation process. Examples included challenges with training^12^ and resourcing. Controlled drugs featured in a number of the subthemes due to the change in workflow for the nurses and the placement of controlled drugs in the ADC. The main issues were with the housing of controlled drugs in the ADC which mostly impacted the pharmacy team resulting in the process of dispensing controlled drugs taking longer than previous. Alanazi et al. (2022)^29^ found that pharmacists in Saudi Arabia reported reduced dispensing times with ADCs but this was not broken down into types of medications. McCarthy et al. (2016)^30^ recognise the benefits that ADCs bring when used in American healthcare settings but strongly advocate for ongoing optimisation post implementation to ensure their full potential is reached. Pederson et al. 2020 recommend ADC optimisation by establishing stock par levels.^31^

Despite there being a number of challenges presented, the participants discussed some of the solutions that occurred to mitigate these challenges. A good example of this was with the training where videos were recorded and added to the intranet for all staff to access. Another solution that resolved communication issues was to implement the weekly meetings between the project team and ensure that meeting notes were documented for later reference and circulated. There were good examples of different teams working together to resolve challenges such as with regards to the scanning of drug barcodes to support the correct placement of medication in the ADC. The recommendations based on the primary findings of this study can be found in Table 1 in the supplementary section.

### 4.1 Strengths and limitations

The first author works in the same department as the pharmacy participants but does not directly work with any of them. The participants may have withheld information because of this.

This study focused on implementation across two sites, covering the reflections of individuals who were part of the core project team and wider implementation team. Hence, providing a view from different perspectives. However, engagement with senior nurses who were part of the implementation team and also from the IT team was difficult. Despite multiple invitations often there was no response, and this led to no participants from IT, although there was one participant from the pharmacy team who specialises in pharmacy IT systems. It is possible that the IT team were short-staffed and therefore could not participate.

Current literature on ADCs reports on their effectiveness on medicines administration and usage. This study provides key information on the barriers to implementation and the enablers for an effective implementation thus supporting others who wish to introduce ADCs or other technologies into healthcare settings. Further evaluation of the long-term success of ADCs is necessary and will look at safety aspects such as availability of medicines, impact on delayed and omitted doses, and the effect of optimisation efforts that have been introduced following this study. Any post implementation evaluation that occurs needs to have a dedicated team to lead improvement projects with evaluation at the end of each improvement cycle.^32^ Standard operating procedures need to be updated with any changes to operational use to ensure staff competence is maintained.

The findings from this study can be used to inform local and national policy with regards to implementation of any technology within the NHS to provide guidance on resource allocation, and scalability.

## 5 Conclusion

The implementation of new technology within the NHS is costly and resource intensive. With appropriate planning and engagement with key stakeholders (pharmacy, nursing leads, estates, IT) at the right time, the implementation can be a success. Without prior planning of stakeholder engagement, implementation leads may find that progress of implementation is delayed. We have identified key barriers and facilitators when implementing ADCs in two busy hospital sites. The main barriers identified were issues related to managing the volume of staff requiring training, the management of controlled drugs in ADCs and problems arising from poor communication. The later introduction of training videos and written material facilitated further training and we recommend that these be available upfront for future technology implementations. Although there were limited facilitators identified to improve the use of controlled drugs within ADCs other than adding them into the ADC at a later point after implementation, the use of regular meetings and documentation reduced communication barriers. We recommend other healthcare organisations to assess the pros and cons carefully when considering putting controlled drugs in ADCs ensuring that nursing colleagues are included in the conversation. We advise to avoid putting liquid controlled drugs in ADCs due to the issues with leaking bottles. Other specific strategies that are recommended are to have nurse staff members as part of the core project team and to introduce mock ADCs to allow staff to become familiar with them prior to full implementation. It is hoped that other healthcare organisations wishing to implement ADCs find this study useful. The learnings of what went well and the solutions implemented should be considered carefully and potentially used, including the recommendations, to adjust strategy to implement ADCs locally. Failure to take these recommendations into account may lead to delays in the progress of ADC implementation, and staff frustration leading to reduced engagement.

## Funding

The authors have no funding to disclose.

## CRediT authorship contribution statement

**Melanie Dalby:** Writing – original draft, Visualization, Validation, Supervision, Software, Resources, Project administration, Methodology, Investigation, Formal analysis, Data curation, Conceptualization. **Ali Alazab:** Writing – review & editing, Validation, Software, Methodology, Investigation, Formal analysis, Data curation. **Navila Talib Chaudhry:** Writing – review & editing, Validation, Supervision.

## Declaration of competing interest

The authors declare that they have no known competing financial interests or personal relationships that could have appeared to influence the work reported in this paper.

## Data Availability

The data that support the findings of this study are available from the corresponding author, [MD], upon reasonable request.

## Appendix A Supplementary data

The following table provides key recommendations for organisations wishing to implement ADCs that emerged during the study. Image 1
