# Supplementary material for: Qualitative analysis to explore the barriers and facilitators around the implementation of automated dispensing cabinets at a large NHS Trust in England
Source: Explor Res Clin Soc Pharm. 2025 Jan 7;17:100562. doi: 10.1016/j.rcsop.2025.100562 (PMC11790501; doi:10.1016/j.rcsop.2025.100562)
Supplement: Supplementary file 1 — The following table provides key recommendations for organisations wishing to implement ADCs that emerged during the study. [file mmc1.docx]

Consolidated criteria for reporting qualitative studies (COREQ): 32-item checklist

| **No** | **Item** | **Guide questions/description** | **Answers from author** |
| --- | --- | --- | --- |
| **Domain 1: Research team and reflexivity** |  |  |  |
| Personal Characteristics |  |  |  |
| 1. | Interviewer/facilitator | Which author/s conducted the interview or focus group? | Ali Alazab, Melanie Dalby |
| 2. | Credentials | What were the researcher's credentials? *E.g. PhD, MD* | Ali Alazab: BPharm, MSc  Melanie Dalby: MPharm, Dip GPP, PG cert IP, PG cert Leadership in Healthcare, PhD |
| 3. | Occupation | What was their occupation at the time of the study? | Ali Alazab: MSc student  Melanie Dalby: Clinical academic research lead for pharmacy |
| 4. | Gender | Was the researcher male or female? | Ali Alazab: Male  Melanie Dalby: Female |
| 5. | Experience and training | What experience or training did the researcher have? | Ali Alazab: Minimal experience but the researcher was supported by the other two authors who have previous experience with focus group and interviews, and previous publications on qualitative research.  Melanie Dalby: Significant experience in qualitative studies. |
| Relationship with participants |  |  |  |
| 6. | Relationship established | Was a relationship established prior to study commencement? | Melanie Dalby knew all the participants prior to the study and works in the same department as them. Ali Alazab did not know any of the participants beforehand. |
| 7. | Participant knowledge of the interviewer | What did the participants know about the researcher? e*.g. personal goals, reasons for doing the research* | Researchers’ role and reasons for conducting the research. |
| 8. | Interviewer characteristics | What characteristics were reported about the interviewer/facilitator? e.g. *Bias, assumptions, reasons and interests in the research topic* | Nil. |
| **Domain 2: study design** |  |  |  |
| Theoretical framework |  |  |  |
| 9. | Methodological orientation and Theory | What methodological orientation was stated to underpin the study? *e.g. grounded theory, discourse analysis, ethnography, phenomenology, content analysis* | Thematic analysis following the six-step process adapted by the framework by Braun & Clarke (2006). |
| Participant selection |  |  |  |
| 10. | Sampling | How were participants selected? *e.g. purposive, convenience, consecutive, snowball* | Purposive sampling with snowballing. |
| 11. | Method of approach | How were participants approached? e*.g. face-to-face, telephone, mail, email* | Via email. |
| 12. | Sample size | How many participants were in the study? | 18 |
| 13. | Non-participation | How many people refused to participate or dropped out? Reasons? | 1 person dropped out, 21 declined or did not respond to the invite. |
| Setting |  |  |  |
| 14. | Setting of data collection | Where was the data collected? e*.g. home, clinic, workplace* | Home (Ali), workplace (Melanie) |
| 15. | Presence of non-participants | Was anyone else present besides the participants and researchers? | No. |
| 16. | Description of sample | What are the important characteristics of the sample? *e.g. demographic data, date* | Mix of female and male and mix of roles. |
| Data collection |  |  |  |
| 17. | Interview guide | Were questions, prompts, guides provided by the authors? Was it pilot tested? | The topic guide was practiced ahead of the first focus group. The topic guide was not provided to the participants. All authors contributed to the topic script. |
| 18. | Repeat interviews | Were repeat interviews carried out? If yes, how many? | No. |
| 19. | Audio/visual recording | Did the research use audio or visual recording to collect the data? | Visual via Microsoft Teams with verbal consent. |
| 20. | Field notes | Were field notes made during and/or after the interview or focus group? | Timings were recorded throughout to help keep to the meeting to time. No other notes were made. |
| 21. | Duration | What was the duration of the interviews or focus group? | The focus group durations ranged from 1 hour and 7 minutes to 1 hour and 58 minutes. The interview durations ranged from 27 minutes to 47 minutes. |
| 22. | Data saturation | Was data saturation discussed? | No. As the sampling was purposive, there was only a specific number of participants that could take part. |
| 23. | Transcripts returned | Were transcripts returned to participants for comment and/or correction? | Some sections were discussed with participants for clarification of meaning and understanding of the recording and transcript. |
| **Domain 3: analysis and findings**z |  |  |  |
| Data analysis |  |  |  |
| 24. | Number of data coders | How many data coders coded the data? | Two. |
| 25. | Description of the coding tree | Did authors provide a description of the coding tree? | No. |
| 26. | Derivation of themes | Were themes identified in advance or derived from the data? | Derived from the data. |
| 27. | Software | What software, if applicable, was used to manage the data? | NVivo version 14.23.3(61) |
| 28. | Participant checking | Did participants provide feedback on the findings? | No. |
| Reporting |  |  |  |
| 29. | Quotations presented | Were participant quotations presented to illustrate the themes / findings? Was each quotation identified? e*.g. participant number* | Yes. |
| 30. | Data and findings consistent | Was there consistency between the data presented and the findings? | Yes. |
| 31. | Clarity of major themes | Were major themes clearly presented in the findings? | Yes. |
| 32. | Clarity of minor themes | Is there a description of diverse cases or discussion of minor themes? | Yes. |
